# Opposing Child Labor

**Published:** 1914-07

**Authors:** 


Opposing Child Labor.—The American Medical Association at its last meeting went on record as opposed to child labor, and decided to use its influence against a practice that is causing so much sickness and early death. There is no doubt that child labor, as it is performed in many States, results in permanent injury to the health of the child, and also makes against virile parenthood. Whatever injures the child today injures the child of tomorrow. Whatever results in a loss of child strength eventually results in a loss of national strength. If this is to be a strong nation, it must have a strong parenthood. In many factory districts there is no such thing as child-life. The children go from babyhood into the responsibilities that should rest only upon mature men and women. When the physicians of the country become thoroughly aroused to the importance of vigorously opposing child labor, the wrongs resulting therefrom will soon stop. It is to be hoped that organized effort may be made to stop the practices that have long been a reproach to our country.

